# Learning Using Concave and Convex Kernels: Applications in Predicting Quality of Sleep and Level of Fatigue in Fibromyalgia

**DOI:** 10.3390/e21050442

**Published:** 2019-04-28

**Authors:** Elyas Sabeti, Jonathan Gryak, Harm Derksen, Craig Biwer, Sardar Ansari, Howard Isenstein, Anna Kratz, Kayvan Najarian

**Affiliations:** 1 Department of Computational Medicine and Bioinformatics, University of Michigan, 2800 Plymouth Rd, NCRC, Ann Arbor, MI 48109-2800, USA; 2 Michigan Center for Integrative Research in Critical Care (MCIRCC), University of Michigan, 2800 Plymouth Rd, NCRC, Ann Arbor, MI 48109-2800, USA; 3 Department of Mathematics, University of Michigan, 2800 Plymouth Rd, Bldg. 18-163, Ann Arbor, MI 48109-2800, USA; 4 Digidence, LLC 7315 Wisconsin Ave., Bethesda, MD 20814-3202, USA; 5 Department of Physical Medicine & Rehabilitation, University of Michigan, 2800 Plymouth Rd, NCRC B14 #D034, Ann Arbor, MI 48109-2800, USA; 6 Department of Emergency Medicine, University of Michigan, 1500 E Medical Center Dr, Ann Arbor, MI 48109, USA; 7 Department of Electrical Engineering and Computer Science, University of Michigan, 1301 Beal Ave, Ann Arbor, MI 48109, USA

**Keywords:** fibromyalgia, Learning Using Concave and Convex Kernels, Empatica E4, self-reported survey

## Abstract

Fibromyalgia is a medical condition characterized by widespread muscle pain and tenderness and is often accompanied by fatigue and alteration in sleep, mood, and memory. Poor sleep quality and fatigue, as prominent characteristics of fibromyalgia, have a direct impact on patient behavior and quality of life. As such, the detection of extreme cases of sleep quality and fatigue level is a prerequisite for any intervention that can improve sleep quality and reduce fatigue level for people with fibromyalgia and enhance their daytime functionality. In this study, we propose a new supervised machine learning method called Learning Using Concave and Convex Kernels (LUCCK). This method employs similarity functions whose convexity or concavity can be configured so as to determine a model for each feature separately, and then uses this information to reweight the importance of each feature proportionally during classification. The data used for this study was collected from patients with fibromyalgia and consisted of blood volume pulse (BVP), 3-axis accelerometer, temperature, and electrodermal activity (EDA), recorded by an Empatica E4 wristband over the courses of several days, as well as a self-reported survey. Experiments on this dataset demonstrate that the proposed machine learning method outperforms conventional machine learning approaches in detecting extreme cases of poor sleep and fatigue in people with fibromyalgia.

## 1. Introduction

Fibromyalgia is medical condition characterized by widespread muscle pain and tenderness that is typically accompanied by a constellation of other symptoms, including fatigue and poor sleep [1,2,3,4,5,6,7,8,9]. Poor sleep, which is a cardinal characteristic of fibromyalgia, is strongly related to greater pain and fatigue, and lower quality of life [10,11,12,13,14,15,16]. As a result, any intervention that can improve sleep quality may enhance daytime functionality and reduce fatigue in people with fibromyalgia.

Studies of sleep in fibromyalgia often rely on self-reported measures of sleep or polysomnography. While easy to administer, self-reported measures of sleep demonstrate limited reliability and validity in terms of their correspondence with objective measures of sleep. In contrast, polysomnography is considered the gold standard of objective sleep measurement; however, it is expensive, difficult to administer, especially on a large scale, and may lack ecological validity. Autonomic nervous system (ANS) imbalance during sleep has been implicated as a mechanism underlying unrefreshed sleep in fibromyalgia. ANS activity can be assessed unobtrusively through ambulatory measures of heart rate variability (HRV) and electrodermal activity (EDA) [17,18]. Wearable devices such as the Empatica E4 are able to directly, continuously, and unobtrusively measure autonomic functioning such as EDA and HRV [19,20,21,22].

In the literature, there are few studies in which machine learning methods are used for classification or prediction of conditions related to fibromyalgia, none of which use physiological signals. A recent survey paper [23] summarizes various types of machine learning methods that have been used in pain research, including fibromyalgia. Previously, using data from 26 individuals (14 individuals with fibromyalgia and 12 healthy controls), the relative performance of machine learning methods for classification of individuals with and without pain using neuroimaging and self-reported data have been compared [24]. In another study using MRI images of 59 subjects, support vector machine (SVM) and decision tree models were used to first distinguish healthy control patients from those with fibromyalgia or chronic fatigue syndrome, and then differentiate fibromyalgia from chronic fatigue syndrome [25]. In [26], an SVM trained on fMRI images was used to distinguish fibromyalgia patients from healthy controls. The combination of fMRI with multivariate pattern analysis has also been investigated in classifying fibromyalgia patients, rheumatoid arthritis patients and healthy controls [27]. Psychopathologic features within an ADABoost classifier have also been employed for classification of patients with fibromyalgia and arthritis [28]. In another recent work [29], secondary analysis of gene expression data from 28 patients with fibromyalgia and 19 healthy controls was used to distinguish between these two groups.

In this study our immediate interest is to predict extreme cases of fatigue and poor sleep in people with fibromyalgia. For such an analysis, we use self-reported quality of sleep and fatigue severity, continuously collected data from the Empatica E4, to measure autonomic nervous system activity during sleep (Section 2). These signals are preprocessed to remove noise and other artifacts as described in Section 3.1. After preprocessing, a number of mathematical features are extracted, including various statistics, signal characteristics, and HRV features (Section 3.2). Section 4 provides a detailed description of our novel Learning Using Concave and Convex Kernels (LUCCK) machine learning method. This model, along with other conventional machine learning methods, were trained on the extracted features and used to predict extreme cases of poor sleep and fatigue, with our method yielding the best results (Section 5).

We believe this analytical framework can be readily extended to outpatient monitoring of daytime activity, with applications to assessing extreme levels of fatigue and pain, such as those experienced by patients undergoing chemotherapy.

## 2. Dataset

The data used for this study was collected from a group of 20 adults with fibromyalgia and consists primarily of a set of signals recorded by an Empatica E4 wristband over the course of seven days (removing 1 h/day for charging/download). Most (80%) participants were female with mean age = 38.79 (min-max = 18–70 years). Of a possible 140 nights of sleep data, the sample had data for 119 (85%) nights. In this dataset, 19.9% of heartbeats were missing due to noisy signals or failure of the Empatica E4 in detecting beats. Data were divided into 5-min windows for HRV analysis; windows with more than 15% missing peaks were eliminated. This led to the exclusion of 30.9% of the windows. The signals used in this analysis are each patient’s blood volume pulse (BVP), 3-axis accelerometer, temperature, and EDA. In addition to these recordings, each subject self-reported his or her wake and sleep times, as well as self-assessed his or her level of fatigue and quality of sleep every morning. These data are labeled by self-reported quality of sleep (1 to 10, 1 being the worst) and level of fatigue (from 1 to 10, 10 indicating the highest level of fatigue).

## 3. Signal Processing: Preprocessing, Filtering, and Feature Extraction

**Figure 1:**
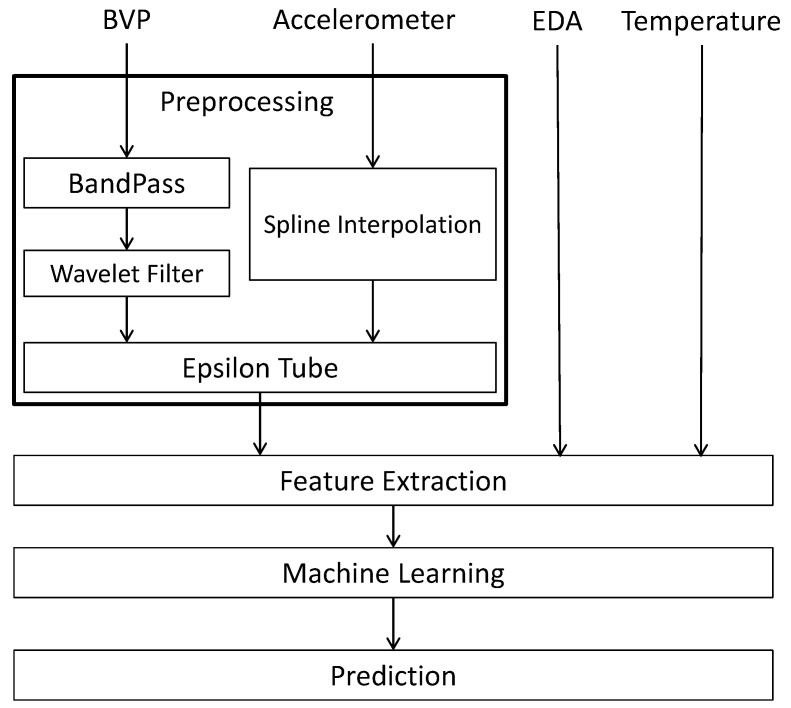
Schematic Diagram of the Proposed Processing System for BVP, accelerometer, EDA and temperature signals.

The schematic diagram of Figure 1 represents our approach to analyzing the BVP and accelerometer signals in the fibromyalgia dataset. During preprocessing, we remove noise from the input signals and format them for future processing (via the Epsilon Tube filter). Once the BVP and accelerometer signals are fully processed, they along with the EDA and temperature signals can then be analyzed and features can be extracted, which in turn leads to the application of machine learning. The final output is a prediction model to which new data can be fed.

### 3.1. Preprocessing

To begin, the raw signals are extracted per patient according to his or her reported wake and sleep times. These are then split into two groups: awake and asleep. For each patient and day, the awake data is paired with the following night’s data and ensuing morning’s self-assessed level of fatigue and quality of sleep.

Our approach to preprocessing BVP signals consists of a bandpass filter (to remove both the low-frequency components and the high-frequency noise), a wavelet filter (to help reduce motion artifacts while maintaining the underlying rhythm), and Epsilon Tube filtering. In order to least perturb the true BVP signal, we chose the Daubechies mother wavelet of order 2 (’db2’) as it closely resembles the periodic shape of the BVP signal. Other wavelets were also considered but ultimately discarded. Once we selected a mother wavelet, we performed an eight-level deconstruction of the input BVP signal. By setting threshold values for each level of detail coefficients (Table 1) and using the results to reconstruct the original signal, we were able to significantly reduce the amount of noise present without compromising the measurement integrity of the underlying physiological values. Utilizing this filter on a number of test cases showed that the threshold values produced consistently useful results regardless of the input, meaning tailored interactions are not required for each signal.

The accelerometer data was upsampled from 32 Hz to 64 Hz via spline interpolation to match the sampling frequency of the BVP signal. The other signals (temperature and EDA) were left unfiltered. We then use these preprocessed signals as input into our main filtering approach (Epsilon Tube), the output of which is then used for feature extraction (Section 3.2).

After filtering of the BVP signal and interpolation of the accelerometer signal, the Epsilon Tube filter [30] is the final component of the preprocessing stage. As discussed in [30], since the BVP signal (and generally any impedance-plethysmography-based measurements) is very susceptible to motion artifact, reduction of this noise is a crucial part of the filtering process. This method uses the synchronized accelerometer data to estimate the motion artifact of BVP signal while leaving the periodic component intact. Let $b_{t}$ represent BVP values at time *t*, *A* a matrix whose rows are the accelerometer signals, and w the vector of Epsilon Tube filter coefficients. Given the tube radius ϵ, the error of $b_{t}$ estimation, i.e., $y_{t}\left(A,w\right)$, is zero if the point $b_{t}$ falls inside the tube

$$|b_{t}-y_{t}{\left(A,w\right)|}_{\epsilon }=\max\{0,|b_{t}-y_{t}\left(A,w\right)\left|-\epsilon \right\}.$$

The Epsilon Tube filter is formulated as a constrained optimization problem that can be expressed as

(1) $$\min\sum_{t=0}^{N-1}\zeta_{t}+\sum_{t=0}^{N-1}\zeta_{t}^{{}^{\prime }}-cR\left(s,A,w\right);$$

subject to

$$\begin{array}{c} b_{t}-y_{t}\left(A,w\right)\leq \epsilon +\zeta_{t}\;t=0,...,N-1;\\ y_{t}\left(A,w\right)-b_{t}\leq \epsilon +\zeta_{t}^{{}^{\prime }}\;t=0,...,N-1;\\ \zeta_{t}\geq 0,\;\zeta_{t}^{{}^{\prime }}\geq 0\;t=0,...,N-1; \end{array}$$

where *N* is the length of BVP signal, $\zeta_{t}$ and $\zeta_{t}^{{}^{\prime }}$ are slack variables, R(s,A,w) is the regularization term and *c* is a designated parameter that adjusts the trade-off between the two objectives. More information about the Epsilon Tube filter can be found in [30]. Taking both the BVP and accelerometer signals as input, the method assumes periodicity in the BVP signal and looks for a period of inactivity at the beginning of the data to use as a template for the rest of the signal. To achieve this, the calmest section of the accelerometer signal (as determined by the longest stretch during which the values never exceed one standard deviation from the mean of the signal) is found. The signal is then shifted so this period of inactivity is at the beginning, and the BVP signal is also shifted to ensure the timestamps remain aligned. The shifted signals are then fed into the Epsilon Tube algorithm, and the resulting output is used for feature extraction.

### 3.2. Feature Extraction

Once the BVP and accelerometer signals are processed, the full signal set is used for feature extraction. There are 91 features extracted from each of the following signals:

- Denoised (filtered) BVP signal, i.e., the output of the Epsilon Tube algorithm, with sampling frequency of 64 Hz.
- Low-band, mid-band, and high-band pass filters applied to the denoised BVP signal.
- Interpolated accelerometer signal, from 32 HZ to 64 Hz.
- Tube sizes from the Epsilon Tube filtering method, another output of the Epsilon Tube algorithm that has the time-varying tube size signal.
- Temperature signal, with sampling frequency of 4 Hz.
- EDA signal, with sampling frequency of 4 Hz.
- The calculated breaths per minute (BPM) signal based on the denoised BVP signal.
- The calculated HRV signal based on the denoised BVP signal.

The extracted features are listed in Table 2. These are extracted from both the awake and the sleep signals, resulting in a full feature set consisting of 182 features. When feature selection is performed using Weka’s information gain algorithm [31] on the first four subjects, the only feature ranked consistently near the top is the average of the BVP signal after being run through a mid-band bandpass filter.

## 4. Machine Learning: Learning Using Concave and Convex Kernels

The final step in the analysis pipeline is the creation of a model that can be used to predict the extreme cases of quality of sleep or level of fatigue for people with fibromyalgia. As detailed in Section 5, in addition to testing a number of conventional machine learning methods, we tested a novel supervised machine learning called Learning Using Concave and Convex Kernels (LUCCK). A key factor in the classification of complex data is the ability of the machine learning algorithm to use vital, feature-specific information to detect settled and complex patterns of changes in the data. The LUCCK method does this by employing similarity functions (defined below) to capture and quantify a model for each of the features separately. The similarity functions are parametrized so that the concavity or convexity of the function within the feature space can be modified as desired. Once the similarity functions and attendant parameters are chosen, the model uses this information to reweight the importance of each feature proportionally during classification.

### 4.1. Notation

In this section, $x\in R^{n}$ is a real-valued vector of features such that $x=(x_{1},\cdots ,x_{n})$, and $x_{i}$ is a real-valued (scalar) feature. Throughout this section, we consider *d* classes, *n* features and *m* (data) samples; also the indexes k=1,⋯,d; i=1,⋯,n; and j=1,⋯,m are used for classes, features and samples respectively. Additionally, $j=1,\cdots ,m_{k}$ refers to $m_{k}<m$ samples in class $C_{k}$.

### 4.2. Classification Using a Similarity Function

An instructive model for comparison to the Learning Using Concave and Convex Kernels method is the *k*-nearest neighbors algorithm [33,34,35] and weighted *k*-nearest neighbors algorithm [36]. In *k*-nearest neighbors, a test sample x is classified by comparing it to the *k* nearest training samples in each class. This can make the classification sensitive to a small subset of samples. Instead, LUCCK classifies test data by comparing it to *all* training data, properly weighted according to their distance to x, which is determined by a similarity function. One major difference between LUCCK and weighted *k*-nearest neighbors is that our approach is based on a similarity function that can be highly non-convex. A fat-tailed (relative to a Gaussian) distribution is more realistic for our data, given that there is a small but non-negligible chance that large errors may occur during measurement, resulting in a large deviation in the values of one or more of the features. The LUCCK method allows for large deviations in a few of the features with only a moderate penalty. Methods based on convex notions of similarity or distance (such as the Mahalanobis distance) are unable to deal adequately with such errors.

Suppose that the feature space is comprised of real-valued vectors $x\in R^{n}$. A *similarity function* is a function $Q:R^{n}\to R$ that measures the closeness of x to the origin, and satisfies the following properties:

1. Q(x)>0 for all $x\in R^{n}$;
2. Q(x)=Q(-x) for all $x\in R^{n}$;
3. Q(λx)>Q(x) if $x\in R^{n}$ is non-zero and |λ|<1.

The value Q(x-y) measures the closeness between the vectors x and y. Using the similarity function Q(x), a classification algorithm can be created as follows:

The set of training data *C* is a subset of $R^{n}$ and is a disjoint union of *d* classes: $C=C_{1}\cup C_{2}\cup \cdots \cup C_{d}$. Let m=|C| be the cardinality of *C* and define $m_{k}=\left|C_{k}\right|$ for all *k* so that $m=m_{1}+\cdots +m_{d}$. To measure the proximity of a feature vector x to a set *Y* of training samples, we simply add the contributions of each of the elements in *Y*:

(2) $$R\left(x,Y\right)=\sum_{y\in Y}Q\left(x-y\right).$$

A vector x is classified in class $C_{k}$, where *k* is chosen such that $R(x,C_{k})$ is maximal. This classification approach can also be used as the maximum a posteriori estimation (details can be found in Appendix A).

### 4.3. Choosing the Similarity Function

The function Q(x) has to be chosen carefully. Let Q(x) be defined as the product

(3) $$Q\left(x\right)=\prod_{i=1}^{n}Q_{i}\left(x_{i}\right),$$

where $x=\left(x_{1},\cdots ,x_{n}\right)\in R^{n}$ and $Q_{i}\left(x_{i}\right)$ only depends on the *i*-th feature. The function $Q_{i}\left(x_{i}\right)$ is again a similarity function satisfying the properties $Q_{i}\left(-x_{i}\right)=Q_{i}\left(x_{i}\right)>0$ for all x∈R, and Q(x)>Q(y) whenever |x|<|y|. After normalization, the $Q,Q_{1},Q_{2},\cdots ,Q_{n}$ can be considered as probability density functions. As such, the product formula can be interpreted as instance-wise independence for the comparison of training and test data. In the naive Bayes method, features are assumed to be independent globally [37]. Summing over all instances in the training data allows for features to be independent in our model.

Next we need to choose the functions $Q_{1},\cdots ,Q_{n}$. One could choose $Q_{i}\left(x_{i}\right)=e^{-\gamma_{i}x^{2}}$, so that

$$Q\left(x\right)=e^{-(\gamma_{1}x_{1}^{2}+\cdots +\gamma_{n}x_{n}^{2})}$$

is a Gaussian kernel function (up to a scalar). However, this does not work well in practice:

- One or more of the features is prone to large errors —The value of Q(x-y) is close to 0 even if x and y only differ significantly in a few of the features. This choice of Q(x) is therefore very sensitive to small subsets of bad features.
- The curse of dimensionality—For the training data to properly represent the probability distribution function underlying the data, the number of training vectors should be exponential in *n*, the number of features. In practice, it usually is much smaller. Thus, if x is a test vector in class $C_{k}$, there may not be a training vector y in $C_{k}$ for which Q(x-y) is not small.

Consequently, let

(4) $$Q_{i}\left(x_{i}\right)={\left(1+\lambda_{i}x^{2}\right)}^{-\theta_{i}},$$

for some parameters $\lambda_{i},\theta_{i}>0$. The function $Q_{i}\left(x_{i}\right)$ can behave similarly to the Cauchy distribution. This function has a “fat tail": as x→∞ the rate that $Q_{i}\left(x_{i}\right)$ goes to 0 is much slower than the rate at which $e^{-\gamma_{i}x^{2}}$ goes to 0. We have

(5) $$Q\left(x\right)=\prod_{i=1}^{n}{\left(1+\lambda_{i}x_{i}^{2}\right)}^{-\theta_{i}}.$$

The function *Q* has a finite integral if $\theta_{i}>\frac{1}{2}$ for all *i*, though this is not required. Three examples of this function can be found in Appendix B.

### 4.4. Choosing the Parameters

Values for the parameters $\lambda_{1},\lambda_{2},\cdots ,\lambda_{n}$ and $\theta_{1},\theta_{2},\cdots ,\theta_{n}$ must be chosen to optimize classification performance. The value of $\log\left(Q_{i}\left(x_{i}\right)\right)=-\theta_{i}\log\left(1+\lambda_{i}x^{2}\right)$ is the most sensitive to changes in *x* when

$$\frac{\partial }{\partial x}\log\left(1+\lambda_{i}x^{2}\right)=\frac{2\lambda_{i}x}{1+\lambda_{i}x^{2}}$$

is maximal. An easy calculation shows that this occurs when $x=\lambda_{i}^{-\frac{1}{2}}$. Since the value $\lambda_{i}$ directly controls the wideness of $Q_{i}\left(x_{i}\right)$’s tail, it is reasonable to choose a value for $\lambda_{i}^{-\frac{1}{2}}$ that is close to the standard deviation of the *i*-th feature. Suppose that the set of training vectors is

$$C=\left\{x^{\left(1\right)},x^{\left(2\right)},\cdots ,x^{\left(m\right)}\right\}\subseteq R^{n},$$

where $x^{\left(j\right)}=\left(x_{1}^{\left(j\right)},\cdots ,x_{n}^{\left(j\right)}\right)$ for all *j*.

Let $s=(s_{1},\cdots ,s_{n})$, where

$$s_{i}=\mathrm{std}\left(x_{i}^{\left(1\right)},x_{i}^{\left(2\right)},\cdots ,x_{i}^{\left(m\right)}\right)$$

be the standard deviation of the *i*-th feature. Let

$$\lambda_{i}=\frac{\Lambda }{s_{i}^{2}}$$

where Λ is some fixed parameter.

Next we choose the parameters $\theta_{1},\cdots ,\theta_{n}$. We fix a parameter Θ that will be the average value of $\theta_{1},\cdots ,\theta_{n}$. If we use only the *i*-th feature, then we define

$$R_{i}\left(x,Y\right)=\sum_{y\in Y}{\left(1+\lambda_{i}{\left(x_{i}-y_{i}\right)}^{2}\right)}^{-\Theta }$$

for any set *Y* of feature vectors. For x in the class $C_{k}$, $\frac{1}{m_{i}-1}R_{i}\left(x,C_{k}\setminus \left\{x\right\}\right)$ gives the average value of ${\left(1+\lambda_{i}{\left(x_{i}-y_{i}\right)}^{2}\right)}^{-\Theta }$ over $y\in C_{k}\setminus \left\{x\right\}$. The quantity $\frac{1}{m_{k}-1}R_{i}\left(x,C_{k}\setminus \left\{x\right\}\right)-\frac{1}{m-1}R_{i}\left(x,C\setminus \left\{x\right\}\right)$ measures how much closer $x_{i}$ is to samples in the class $C_{k}$ than to vectors in the set *C* of all feature vectors except x itself. This value measures how well the *i*-th feature can classify x as lying in $C_{k}$ as opposed to some other class. If we sum over all x∈C and ensure that the result is non-negative we obtain

(6) $$\begin{array}{c} \alpha_{i}=\max\left\{0,\sum_{k=1}^{d}\sum_{x\in C_{k}}\left(\frac{R_{i}\left(x,C_{k}\setminus \left\{x\right\}\right)}{m_{k}-1}-\frac{R_{i}\left(x,C\setminus \left\{x\right\}\right)}{m-1}\right)\right\}. \end{array}$$

The $\theta_{1},\cdots ,\theta_{n}$ can be chosen so that they have the same ratios as $\alpha_{1},\cdots ,\alpha_{n}$ and sum up to nΘ:

(7) $$\theta_{i}=\frac{n\alpha_{i}\Theta }{\sum_{i=1}^{n}\alpha_{i}}.$$

In terms of complexity, if *n* is the number of features and *m* is the number of training samples then the complexity of the proposed method would be $O(n\times m^{2})$.

### 4.5. Reweighting the Classes

Sometimes a disproportionate number of test vectors are classified as belonging to a particular class. In such cases one might get better results after reweighting the classes. The weights $\omega_{1},\omega_{2},\cdots ,\omega_{d}$ can be chosen so that all are greater than or equal to 1. If *p* is a probability vector, then we can reweight it to a vector

$$W_{\omega }\left(p\right)=\left(p_{1}^{\prime },\cdots ,p_{d}^{\prime }\right)$$

where

$$p_{l}^{\prime }=\frac{\omega_{l}p_{l}}{\sum_{k=1}^{d}\omega_{k}p_{k}}.$$

If the output of the algorithm consists of the probability vectors $p\left(x^{\left(1\right)}\right),\cdots ,p\left(x^{\left(m\right)}\right)$ the algorithm can be modified so that it yields the output $W_{\omega }\left(p\left(x^{\left(1\right)}\right)\right),\cdots ,W_{\omega }\left(p\left(x^{\left(m\right)}\right)\right)$. A good choice for the weights $\omega_{1},\cdots ,\omega_{d}$ can be learned by using a portion of the training data. To determine how well a *training* vector x∈C can be classified using the remaining training vectors in C∖{x}, we define

$${\tilde{p}}_{k}\left(x\right)=\frac{R(x,C_{k}\setminus \left\{x\right\})}{R(x,C\setminus \{x\})}.$$

The value ${\tilde{p}}_{k}\left(x\right)$ is an estimate for the probability that x lies in the class $C_{k}$, based on all feature vectors in *C* except x itself. We consider the effect of reweighting the probabilities ${\tilde{p}}_{k}\left(x\right)$, by

$${\tilde{p}}_{k}^{\prime }\left(x\right)=\frac{\omega_{i}\tilde{p}\left(x\right)}{\sum_{i=1}^{d}\omega_{i}{\tilde{p}}_{i}\left(x\right)}.$$

If x lies in the class $C_{k}$, then the quantity

$$\max\left\{{\tilde{p}}_{1}^{\prime }\left(x\right),\cdots ,{\tilde{p}}_{d}^{\prime }\left(x\right)\right\}-{\tilde{p}}_{k}^{\prime }\left(x\right)$$

measures how badly x is misclassified if the reweighting is used. The total amount of misclassification is

$$\begin{array}{c} \sum_{k=1}^{d}\sum_{x\in C_{k}}\left(\max\left\{{\tilde{p}}_{1}^{\prime }\left(x\right),\cdots ,{\tilde{p}}_{d}^{\prime }\left(x\right)\right\}-{\tilde{p}}_{k}^{\prime }\left(x\right)\right)=\\ \sum_{k=1}^{d}\sum_{x\in C_{k}}\left(\frac{\max\left\{\omega_{1}{\tilde{p}}_{1}\left(x\right),\cdots ,\omega_{d}{\tilde{p}}_{d}\left(x\right)\right\}-\omega_{k}{\tilde{p}}_{k}\left(x\right)}{\sum_{l=1}^{d}\omega_{l}{\tilde{p}}_{l}\left(x\right)}\right). \end{array}$$

We would like to minimize this over all choices of $\omega_{1},\cdots \omega_{d}\geq 1$. As this is a highly nonlinear problem, making optimization difficult, we instead minimize

$$\begin{array}{c} \sum_{k=1}^{d}\sum_{x\in C_{k}}\left(\max\left\{\omega_{1}{\tilde{p}}_{1}\left(x\right),\cdots ,\omega_{d}{\tilde{p}}_{d}\left(x\right)\right\}-\omega_{k}{\tilde{p}}_{k}\left(x\right)\right)=\\ \sum_{x\in C}\max\left\{\omega_{1}{\tilde{p}}_{1}\left(x\right),\cdots ,\omega_{d}{\tilde{p}}_{d}\left(x\right)\right\}-\sum_{k=1}^{d}\omega_{k}\sum_{x\in C_{k}}{\tilde{p}}_{k}\left(x\right). \end{array}$$

instead. This minimization problem can be solved using linear programming, i.e., by minimizing the quantity

$$\sum_{j=1}^{m}z^{\left(j\right)}-\sum_{k=1}^{d}\omega_{k}\sum_{x\in C_{k}}{\tilde{p}}_{k}\left(x\right).$$

for the variables $\omega_{1},\cdots ,\omega_{d}$ and new variables $z^{\left(1\right)},\cdots ,z^{\left(m\right)}$ under the constraints that

$$z^{\left(j\right)}\geq \omega_{k}\tilde{p}\left(x^{\left(j\right)}\right)$$

and

$$\omega_{k}\geq 1$$

for all *k* and *j* with 1≤k≤d and 1≤j≤m.

## 5. Experiments

In this section, the performance of LUCCK is first compared with other common machine learning methods using four conventional datasets, after which its performance on the fibromyalgia dataset is evaluated.

### 5.1. UCI Machine Learning Repository

In this set of experiments, LUCCK in compared to some well-known classification methods on a number of datasets downloaded from the University of California, Irvine (UCI) Machine Learning Repository [38]. Each method was tested on each dataset using 10-fold cross-validation, with the average performance and execution time across all folds provided in Table 3. Table 4 contains the average values for accuracy and time across all four datasets.

### 5.2. Fibromyalgia Dataset

In this study, we have created a model that can be used to predict the quality of sleep or level of fatigue for people with fibromyalgia. The labels are self-assessed scores ranging from 1 to 10. Attempts to develop a regression model showed less promise than the results from a binary split. The most likely reason for this failure of the linear regression model is the nature of self-reported scores, especially those related to patient assessment of their level of pain. This fact is primarily due to the differences in individual levels of pain-tolerance. In previous studies [39,40], proponents of neural "biomarkers" argued that self-reported scores are unreliable, making objective markers of pain imperative. In another study [24], self-reported scores were found to be reliable only for extreme cases of pain and fatigue. Consequently, in this study, binary classification of extreme cases of fatigue and poor sleep is investigated. In this situation, a cutoff value is selected: patients that chose a value less than the threshold are placed in one group, while those that chose a value above the threshold are placed in another. As such, the values >8 are chosen for extreme cases of fatigue, and the values <4 are chosen for extreme cases of poor sleep quality. In this way, binary classifications are possible (>8 vs. <8 for fatigue and >4 vs. <4 for sleep). Using the extracted feature set, machine learning algorithms are applied and tested using 10-fold cross-validation. This is done in a way so as to prevent the data from any one patient being in multiple folds: all of a given patient’s data are including entirely in a single fold. In addition, in order to address possibly imbalanced data during fold creation, random undersampling is performed to ensure the ratio between the two classes is not less than 0.3 (this rate is chosen since the extreme cases are at most 30 percent of the [1,10] interval of self-reported scores). This prevents the methods from developing a bias towards the larger class.

#### 5.2.1. Results with Conventional Machine Learning Methods

A number of conventional machine learning models listed in Table 5 were applied to the extracted data in this study. As can be seen, many major machine learning methods were tested. For each of these methods, various configurations were tested, and the best sets of parameters were chosen using cross-validation (hyperparameter optimization). For instance, we used the combination of AdaBoost with different types of standard methods such as Decision Stump and Random Forest in order to explore the possibility of improving the performance of these methods via boosting. The *k*-nearest neighbor method with k=7 was used in this experiment. For the weighted *k*-nearest neighbor method [36], the inversion kernel (inverse distance weights) with k=7 resulted in the best performance. For the Neural Network algorithm, the Weka (Waikato Environment for Knowledge Analysis) [41] multilayer perceptron with two hidden layers was used. The results of using these machine learning approaches for prediction of extreme sleep quality (cutoff of 4) and fatigue level (cutoff of 8) are presented in Table 5. As shown in this table, the AdaBoost method based on random forest yielded the best results for quality of sleep (based on area under the receiver operating characteristic curve, or AUROC). For level of fatigue, the neural network was the best performing model.

#### 5.2.2. Results with Our Machine Learning Method: Machine Learning Using Concave and Convex Kernels

In addition to the aforementioned conventional methods, we also used our machine learning approach that resulted in superior performance compared to the standard machine learning methods discussed above. Recall that in the Learning Using Concave and Convex Kernels algorithm, test data is classified by comparing it to all training data, properly weighted according to information extracted from each of the features (see Section 4 for further details). The results of applying our method to fibromyalgia are presented in Table 5, with cutoff values of 4 and 8 for quality of sleep and level of fatigue, respectively. As can be seen, LUCCK was able to vastly outperform other models on the fatigue outcome; however, the improvement on sleep outcome was not significant. This disparity is likely due to the different feature spaces for the sleep and fatigue outcomes. In general, the feature space for fatigue is significantly more dispersed, due to there being more samples (during daytime) and also that daytime activity negatively affects the signal quality, increasing dispersion. In contrast, signals (and their associated features) recorded during sleep are of better quality. This leads to the better prediction result for sleep in all methods used. Our proposed LUCCK algorithm can ameliorate the nature of the fatigue feature space, as it is specifically designed to reduce the effect of training data for which there is a large deviation from test data. As such, LUCCK was able to vastly outperform other models on the fatigue outcome. We should note that while the cohort size in this study seems to be limited, the continuous recording of physiological signals for seven days and nights created a comprehensive dataset. Additionally, similar to *k*-NN and its weighted version (and unlike SVM and neural network models), LUCKK can be trained even with few samples, which is one advantage of the proposed algorithm.

## 6. Conclusions and Discussion

In this study we primarily focused on prediction of the extreme cases of fatigue and poor sleep. As such, we have created preprocessing/conditioning methods that have the ability to improve the quality of parts of the signals with low quality due to motion artifact and noise. In addition, we identified a set of mathematical features that are important in extracting patterns from physiological signals that can distinguish poor and good clinical outcomes for applications such as fibromyalgia. Additionally, we showed that our proposed machine learning method outperformed the standard methods in predicting the outcomes such as fatigue and sleep quality. Generally, our proposed framework (preprocessing, mathematical features, and proposed machine learning method) can be employed in any study that involves prediction using BVP, HRV and EDA signals.

The epsilon tube filter is covered by US Patent 10,034,638, for which Kayvan Najarian is a named inventor.

## Figures and Tables

**Table 1:** Chosen coefficient thresholds for the 8-level wavelet decomposition.

Detail Coefficients Level	Threshold
8	94.38
7	147.8
6	303.1
5	329.9
4	90.16
3	30.67
2	0
1	0

**Table 2:** The list of features extracted from all signals.

Signals	Features
Denoised BVP	Mean, Standard deviation, Variance, Power, Median, Frequency with the highest peak,
	Amplitude of the frequency with highest peak, FFT power, Mean of FFT amplitudes,
	Mean of the FFT frequencies, Median of FFT amplitudes (11 features)
Low-band denoised	Mean, Standard deviation, Variance, Power, Median, Frequency with the highest peak,
BVP	Amplitude of the frequency with highest peak, FFT power, Mean of FFT amplitudes,
	Mean of the FFT frequencies, Median of FFT amplitudes (11 features)
Mid-band denoised	Mean, Standard deviation, Variance, Power, Median, Frequency with the highest peak,
BVP	Amplitude of the frequency with highest peak, FFT power, Mean of FFT amplitudes,
	Mean of the FFT frequencies, Median of FFT amplitudes (11 features)
High-band denoised	Mean, Standard deviation, Variance, Power, Median, Frequency with the highest peak,
BVP	Amplitude of the frequency with highest peak, FFT power, Mean of FFT amplitudes,
	Mean of the FFT frequencies, Median of FFT amplitudes (11 features)
Tube size	Mean, Standard Deviation, Variance, Power (4 features)
Interpolated	Mean, Standard Deviation, Variance, Power (4 features)
accelerometer	
Temperature signal	Mean, Standard Deviation, Variance, Power (4 features)
EDA signal	Mean, Standard Deviation, Variance, Power (4 features)
BPM signal	Maximum, Minimum, Range, Mean, Standard deviation, Power (6 features)
HRV	The Kubios Standard HRV feature set [32] (25 features)

**Table 3:** Comparison of our proposed method (LUCCK) with other machine learning methods in terms of accuracy and running time, averaged over 10 folds.

Dataset	Method	Accuracy (%)	Time (s)
Sonar (208 samples)	LUCCK	87.42	1.5082
	3-NN	81.66	0.0178
	5-NN	81.05	0.0178
	Adaboost	82.19	1.0239
	SVM	81.00	0.0398
	Random Forest (10)	78.14	0.1252
	Random Forest (100)	83.39	1.1286
	LDA	74.90	0.0343
Glass (214 samples)	LUCCK	82.56	0.3500
	3-NN	68.72	0.0161
	5-NN	67.04	0.0162
	Adaboost	50.82	0.5572
	SVM	35.57	0.0342
	Random Forest (10)	75.31	0.1062
	Random Forest (100)	79.24	0.9319
	LDA	63.28	0.0155
Iris (150 samples)	LUCCK	95.93	0.1508
	3-NN	96.09	0.0135
	5-NN	96.54	0.0135
	Adaboost	93.82	0.4912
	SVM	96.52	0.0143
	Random Forest (10)	94.81	0.0889
	Random Forest (100)	95.29	0.7686
	LDA	98.00	0.0122
E. coli (336 samples)	LUCCK	87.61	0.5937
	3-NN	85.08	0.0190
	5-NN	86.43	0.0193
	Adaboost	74.13	0.6058
	SVM	87.53	0.0448
	Random Forest (10)	84.56	0.1075
	Random Forest (100)	87.34	0.9265
	LDA	81.46	0.0182

**Table 4:** Model accuracy with standard deviation and execution time for each model, averaged across the four UCI datasets.

Method	Accuracy (%)	Time (s)
LUCCK	88.38 ± 5.55	0.6507
3-NN	82.89 ± 11.27	0.0166
5-NN	82.77 ± 12.29	0.0167
Adaboost	75.24 ± 18.18	0.6695
SVM	75.16 ± 27.15	0.0333
Random Forest (10)	83.21 ± 8.65	0.1070
Random Forest (100)	86.32 ± 6.84	0.9389
LDA	79.41 ± 14.49	0.0201

**Table 5:** Results of conventional machine learning methods.

Method	Sleep		Fatigue	
	Accuracy (%)	AUROC	Accuracy (%)	AUROC
AdaBoost - Decision Stump	62.07	0.63	46.64	0.55
AdaBoost - Random Forest	59.97	0.65	51.24	0.55
K-Nearest Neighbor	60.55	0.55	51.88	0.53
Weighted K-Nearest Neighbor	65.27	0.62	68.05	0.51
Neural Network	63.47	0.64	54.80	0.59
Random Forest	63.32	0.63	52.46	0.57
Support Vector Machine	64.47	0.50	55.94	0.50
LUCCK	66.95	0.66	87.59	0.68

**Figure A1:**
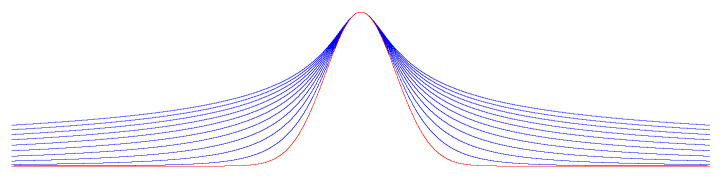
$Q\left(x\right)={\left(1+\lambda_{1}x^{2}\right)}^{-1/\lambda_{1}}$ with for $\lambda_{1}=0.4,0.8,\cdots ,4$ (blue curves) and $\lambda_{1}=0$ (red curve).

**Figure A2:**
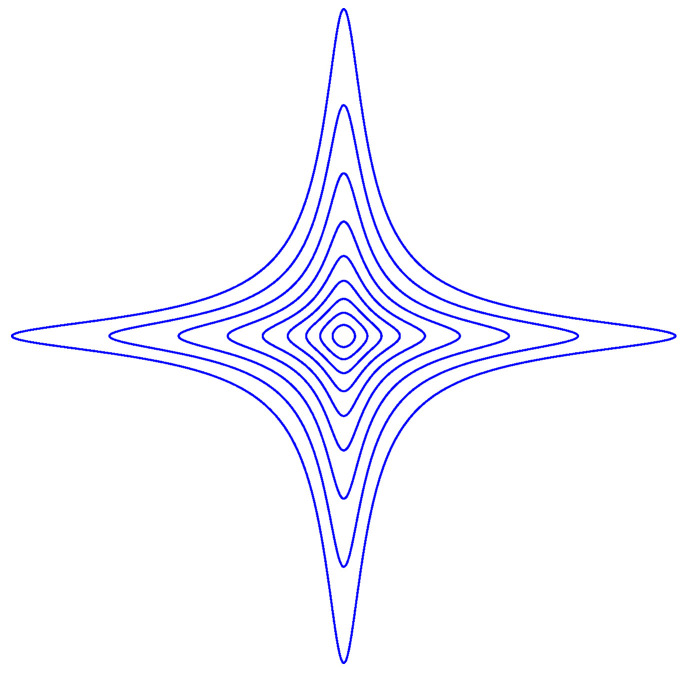
$Q\left(x\right)={\left(1+x_{1}^{2}\right)}^{-1}{\left(1+x_{2}^{2}\right)}^{-1}=\alpha$ with 0<α<1.

**Figure A3:**
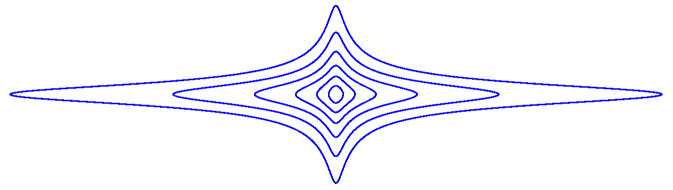
$Q\left(x\right)={\left(1+2x_{1}^{2}\right)}^{-\frac{1}{2}}{\left(1+x_{2}^{2}\right)}^{-1}=\alpha$ with 0<α<1.

## Abbreviations

The following abbreviations are used in this manuscript:

LUCCK	Learning Using Concave and Convex Kernels
BVP	Blood Volume Pulse
EDA	Electrodermal Activity
ANS	Autonomic Nervous System
HRV	Heart Rate Variability
FFT	Fast Fourier transform
BPM	Breaths Per Minute
AUROC	Area Under Receiver Operating Characteristic Curve

## Appendix A Classification as Maximum a Posteriori Estimation

The classification approach suggested in Section 4.2 can also be viewed in terms of probability density functions. Suppose that $\int_{R^{n}}Q\left(x\right)=e$ with 0<e<∞. The function

(A1) $$f_{C}\left(x\right)=\frac{R(x,C)}{me}={\left(me\right)}^{-1}\sum_{y\in C}Q\left(x-y\right)$$

is therefore a probability density function. This probability density function is an estimation for the probability distribution from which the training data were taken.

We have

$$f_{C}=p\left(C_{1}\right)f_{C_{1}}+\cdots +p\left(C_{d}\right)f_{C_{d}}$$

where

(A2) $$f_{C_{k}}\left(x\right)=\frac{R(x,C_{k})}{m_{k}e}={\left(m_{k}e\right)}^{-1}\sum_{y\in C_{k}}Q\left(x-y\right)$$

is a probability density function for the training data in class $C_{k}$ for k=1,2,⋯,d and $p\left(C_{k}\right):=\frac{m_{k}}{m}$ is the probability that a randomly chosen training vector lies in $C_{k}$. $f_{C}$ can be considered as a mixture of the probability density functions $f_{C_{1}},\cdots ,f_{C_{d}}$. Suppose that $x\in R^{n}$ is taken from the distribution $f_{C_{k}}$ with probability $p(C_{k})$, then the distribution for x is $f_{C}$. Given the outcome x, the probability that it was taken from the distribution $f_{C_{k}}$ is

$$p_{k}\left(x\right):=\frac{p\left(C_{k}\right)f_{C_{k}}\left(x\right)}{f_{C}\left(x\right)}=\frac{R(x,C_{k})}{R(x,C)}.$$

This shows that the classifying scheme is the maximum a posteriori estimation. Instead of classifying a feature vector, the probability vector

$$p\left(x\right)=(p_{1}\left(x\right),p_{2}\left(x\right),\cdots ,p_{d}\left(x\right))$$

can be given as output. The formula

$$p_{k}\left(x\right)=\frac{R(x,C_{k})}{R(x,C)}$$

is well-formed, even if Q(x) does not have a finite integral, which may be the case in some examples.

## Appendix B. Examples

**Example** **A1.**
*Suppose that there is only one feature, i.e., n=1, then Q(x) can be defined as*

$$Q\left(x\right)={\left(1+\lambda_{1}x^{2}\right)}^{-\theta_{1}},$$

*whose graph at various values of θ and λ is depicted in Figure A1:*

*As $\lambda_{1}$ goes to zero, the function converges to the normal distribution $e^{-x^{2}}$ (the red curve in Figure A1).*

**Example** **A2.**
*Suppose that n=2, then Q(x) is defined as*

$$Q\left(x_{1},x_{2}\right)={\left(1+x_{1}^{2}\right)}^{-1}{\left(1+x_{2}^{2}\right)}^{-1},$$

*with $\theta_{1}=\theta_{2}=\lambda_{1}=\lambda_{2}=1$. Q(x) is depicted in Figure A2 at various level curves for Q(x)=α, with 0<α<1.*

*The Equation $Q(x_{1},x_{2})=\alpha$ is a closed curve. Such a curve can be thought of as the set of all points that have a given distance to the origin. We observe that for $\alpha \geq \frac{1}{4}$, the neighborhood*

$$\left\{x\in R^{2}|Q\left(x\right)>\alpha \right\}$$

*of the origin is convex, but for $\alpha <\frac{1}{4}$ it is not.*

**Example** **A3.**
*Consider the case when n=2 and $\theta_{1}=1$, $\theta_{2}=2$, $\lambda_{1}=1$ and $\lambda_{2}=\frac{1}{2}$, then Q(x) is defined as*

$$Q\left(x_{1},x_{2}\right)={\left(1+2x_{1}^{2}\right)}^{-\frac{1}{2}}{\left(1+x_{2}^{2}\right)}^{-1}.$$

*Q(x) is depicted in Figure A3 at various level curves for Q(x)=α, with 0<α<1.*

*For small values of x, the function Q is equally sensitive to $x_{1}$ and $x_{2}$. However, if x is large, then Q is more sensitive to $x_{2}$.*
